# Comparison of two response tracking methods for sound localization with concealed sound sources

**DOI:** 10.1038/s41598-026-58759-6

**Published:** 2026-07-15

**Authors:** Franz-Ullrich Müller, Sebastian Roth, Julian Angermeier, Werner Hemmert, Stefan Zirn

**Affiliations:** 1 Department of Electrical Engineering, Medical Engineering and Computer Science, Peter Osypka Institute of medical engineering, NeuroAkustik, Offenburg University of Applied Sciences, Badstraße 24, 77652 Offenburg, Germany; 2 School of Computation, Information and Technology, Bioanalog information processing, Technical University of Munich, 85748 Munich, Germany; 3 Osypka AG, Rheinfelden, Germany

**Keywords:** Binaural hearing, Spatial hearing, Sound localization, Response tracking, Visual bias, Engineering, Neuroscience, Psychology, Psychology

## Abstract

Accurate measurement of sound localization depends not only on the acoustic setup but also on the response method used to report the perceived source direction, particularly when visual cues from loudspeaker arrays are minimized. This study compared two response tracking methods specifically designed for concealed horizontal loudspeaker arrays: An egocentric LED-based method and an exocentric tablet-based method. Both methods are compact and require moderate technical effort compared to high-end solutions such as head- or eye-tracking systems. 20 normal-hearing participants completed free-field sound localization tasks using both approaches. The egocentric LED-based method produced a significantly lower localization error (RMSE: 5.4°, 95% CI: 4.77° to 6.10°) than the tablet-based method (7.4°, 95% CI: 6.75° to 8.07°), but required longer trial durations (21% increase). Most of the difference in RMSE was attributable to the discrepancies between the finger position on the tablet surface and the intended target position. Angle-dependent analyses revealed systematic, method-specific effects: Both response methods exhibited a central shift in participants’ responses and reduced precision at lateral source positions. Exploratory analyses of the tablet-based method suggested the effect was superimposed by an additional and constant lateral bias across the source angles. These findings demonstrate that the choice of response method can systematically influence measured localization performance and should therefore be taken into account when designing localization experiments and interpreting their results.

**Supplementary Information:**

The online version contains supplementary material available at 10.1038/s41598-026-58759-6.

## Introduction

Accurate assessment of human sound localization performance depends not only on the playback setup that presents the stimuli, but also on the response tracking method participants used to indicate the perceived location of a sound source. Over the years, various response-tracking methods have been proposed, which can be broadly classified into exocentric and egocentric methods.

 Exocentric methods require participants to indicate the perceived source location on a two- or three-dimensional representation of the test environment ^1–4^. These methods allow for fast responses that can cover every room direction. However, they are prone to systematic judgment errors ^5^. Participants often struggle to map their auditory environment onto an abstract representation like a sphere, a piece of paper or a computer screen^ 6^, which can compromise localization accuracy.

Most widespread are egocentric methods that require the participants to indicate the sound source directly within the environment, either by selecting a corresponding speaker by label^ 1,7–10^ or by tracking the position of the participant’s hand, head, eyes, body or a handheld object ^11–18^. These approaches generally demonstrate higher precision than exocentric methods ^5,6^. However, positional tracking requires more sophisticated hardware and complex post-processing, while the fidelity of label-based methods is directly tied to the number of sound sources in the playback setup.

In most experimental setups, participants are seated at the center of an array of loudspeakers. Configurations range from (semi-)circular ^3,19,20^ and rectangular setups ^21^, used to assess localization accuracy in the horizontal plane to spherical arrays, able to include elevation as a test parameter ^4,12^. In many studies, the loudspeakers are visible or explicitly labeled, which can introduce a visual bias toward discrete loudspeaker positions. If the angular spacing of the speakers exceeds a participant’s inherent localization error, the localization accuracy will be underestimated if the correct loudspeaker was selected and overestimated if the wrong speaker was chosen. Roth et al. ^22^ showed 2022 that the error in localization accuracy more than doubled when an array of seven loudspeakers was concealed from view of the participants. Reducing the speaker separation to an angle within a participant’s localization error can mitigate this bias. However, this is not always feasible due to spatial and hardware constraints.

The visual bias can be avoided by concealing the loudspeakers, either by conducting the test in darkness ^12^ or by covering the array. Although conducting the test in the dark offers an easy solution, the unfamiliar situation could induce discomfort and potentially impair the spatial orientation of the participant. Covering the speakers, on the other hand, requires additional effort or expenditure to ensure proper signal representation, either by use of opaque acoustic fabric with minimal damping properties or by equalizing the output signal to compensate the dampening introduced by the materials used.

Despite the potential benefits of concealed loudspeaker arrays, the literature on sound-localization methods that minimize visual bias remains limited. Moreover, concealing the loudspeakers precludes simple label-based response strategies and typically necessitates more complex response methods to indicate perceived source locations.

In this context, the present study compared two response tracking methods designed for use with concealed loudspeaker arrays: An egocentric LED-based method and an exocentric tablet-based method. Both methods are compact and require moderate technical effort to implement. The comparison focused on their relative advantages and disadvantages in terms of localization accuracy and trial duration.

## Method

The LED-based and tablet-based response methods were compared in a free-field localization task with concealed loudspeakers.

The study was conducted in accordance with the Code of Ethics of the World Medical Association (Declaration of Helsinki) for experiments involving humans and was approved 2019 by the Technical University of Munich ethics committee (340/19).

### Response methods

**Fig. 1:**
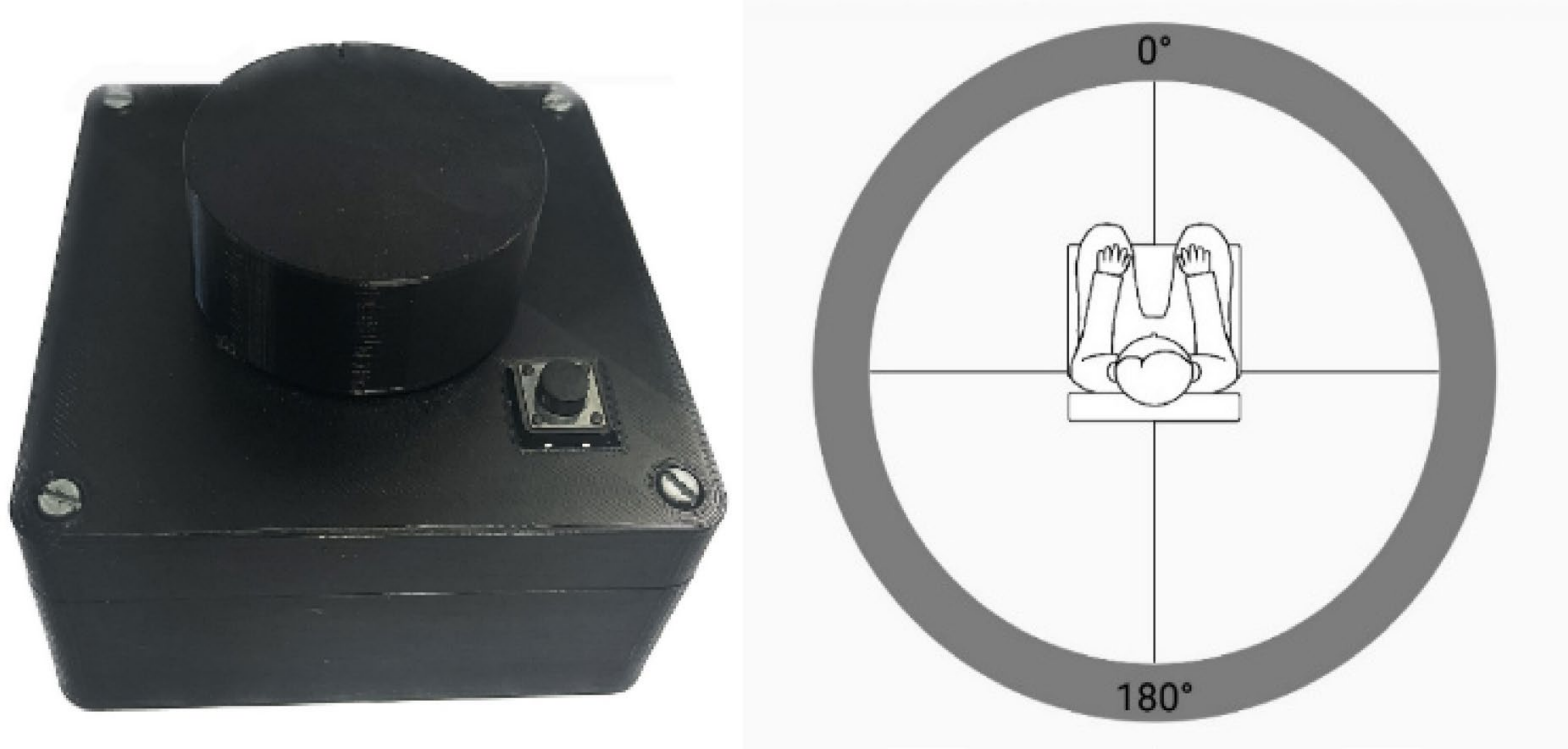
User interfaces of the two response methods: Left: Rotary encoder and pushbutton in a 3D-printed housing ^46^ used for positional control of the lit LED along the array. Right: Graphic user interface of the tablet app showing a schematic representation of the test environment. The gray circle represents the covered speaker array. Responses are tracked by touching the circle at the angle corresponding with the perceived sound source location.

The LED-based method utilized a 3-meter-long LED-strip consisting of 181 individual LEDs. The strip was fixed to a curved metal rail positioned slightly below and directly in front of the loudspeaker array. The setup covered a range of −90° to + 90° providing an angular resolution of 1°. Participants indicated the perceived sound source location by rotating a hand-held rotary encoder, which moved a lit pointer LED along the strip. The selected position of the pointer LED could be confirmed by pressing a pushbutton located next to the rotary encoder (Fig. 1 - left). The rotary encoder, pushbutton, and LED strip were all connected via cable to an Arduino microcontroller, which handled the control logic and transmitted the input data to a PC via USB connection.

The tablet-based method used a 10-inch touchscreen tablet (Samsung Galaxy Tab A SM-T580) displaying a graphical user interface with stylized top-down view of the participant, surrounded by a continuous circle representing the loudspeaker array. The circle had line indicators at 0°, −90°, 90° and 180° and text indicators at 0° and 180° (Fig. 1 - right). Participants responded by tapping the circle at the position corresponding to the perceived sound source direction. Successful input was visually confirmed by a brief animation in which the circle expanded by approximately 2 mm and then returned to its original size within 0.5 s. The coordinates for the centroid of the touched area were provided by the tablet software and directly converted into angle values depending on the pixel position on the circle. The tablet interface provided the same input range (− 90° to + 90°) and angular resolution (1°) as the LED-based method. The tablet was mounted on a stand positioned at chest height of the participant, within comfortable arm’s reach. Input data from the tablet was transmitted to a PC via Bluetooth connection ^23^.

### Test setup

**Fig. 2:**
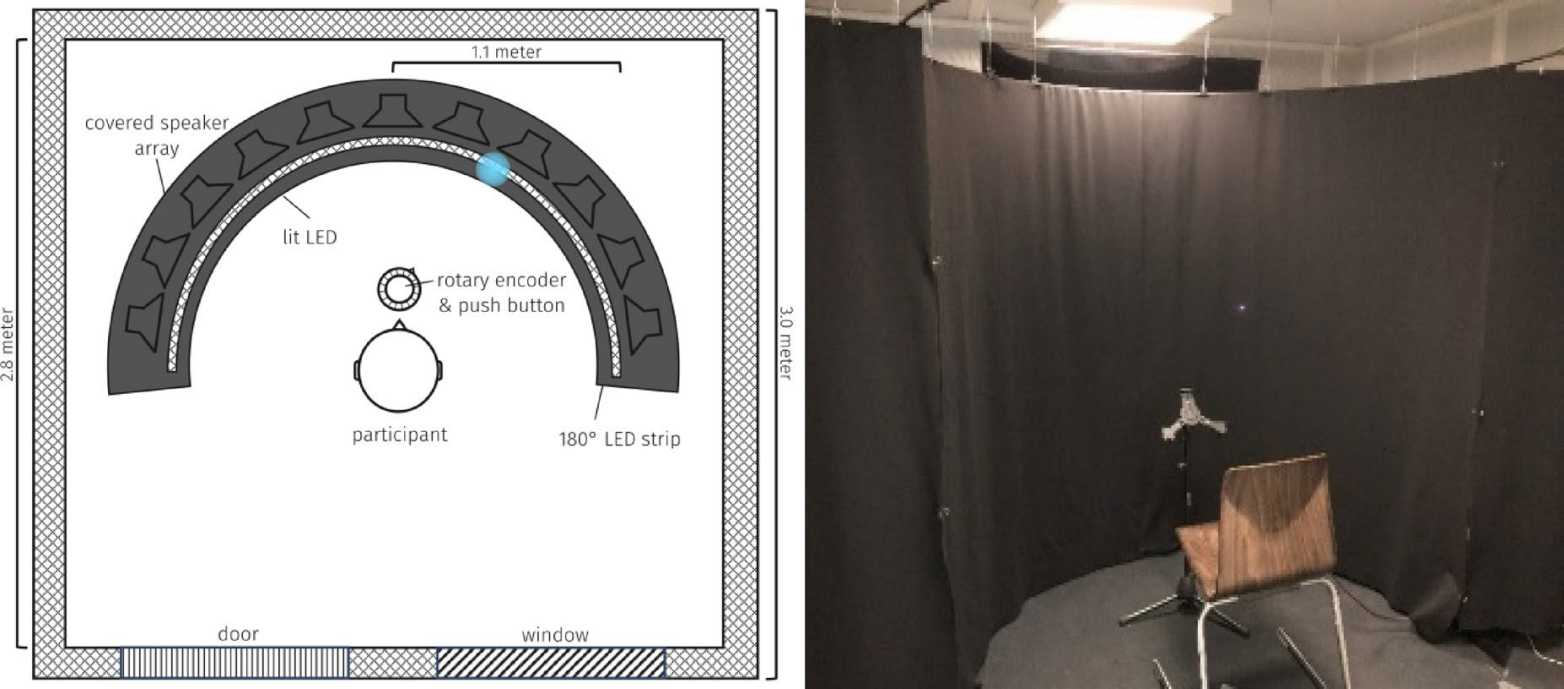
Experimental setup:Schematic illustration (left) and photograph (right) of the concealed loudspeaker array inside the sound-attenuated booth. The photograph shows the suspended acoustic fabric with the indicator LED shiningthrough the material.

The playback setup consisted of eleven loudspeakers (Genelec 8030 C) arranged in an horizontal semicircular configuration inside a 2.8 × 2.8 × 2 m sound-attenuated booth (Fig. 2). The loudspeakers were positioned at 15° intervals, covering an azimuthal range from − 75° to + 75°. The ± 90° positions were intentionally omitted to avoid boundary effects at the limits of the response methods’ input range. The array had a radius of 1.1 m and all loudspeakers were mounted at head height (1.2 m) of the seated participant. The radius was chosen as a compromise between the spatial constraints of the sound-attenuated booth and the requirement to ensure far-field sound propagation at the listener’s position.

To eliminate visual cues, both the loudspeaker array and the LED strip were covered with opaque but translucent acoustic fabric (dampening: −2.3 dB above 2 kHz) suspended from a circular frame mounted to the ceiling of the booth (Fig. 2). The fabric was placed close to the loudspeakers and LED strip to prevent parallax errors, ensuring that the LED lights shining through it aligned with the corresponding speaker positions. The speaker setup was calibrated with a handheld audio and acoustic analyzer (NTi Audio XL2) at the participant’s head position at the height of 1.2 m in the center of the array.

### Participants & task

Twenty normal-hearing adults (eleven female and nine male) between the age of 20 and 26 years (mean: 23 ± 2 years) participated in the experiment. All had audiometric thresholds ≤ 20 dB HL at 0.5, 1, 2, and 4 kHz and reported no neurological or cognitive impairments. Written informed consent was obtained from all participants and participant data were stored under pseudonyms in a secure offline database.

Participants were randomly assigned to one of two groups to counterbalance test order. Each participant completed the localization task once with each response method.

During testing, participants were seated in the center of the loudspeaker array. They were instructed to face a lit pointer LED at 0° during stimulus presentation. Following the stimulus presentation, they were allowed to move their head freely and indicate the perceived sound source direction using the assigned response method. After each response, the pointer LED returned to 0°, and participants had 2 s to reorient before the start of the next trial. Participants were observed via a window in the sound-attenuated booth to ensure task compliance. After completion of the first test run, participants were offered a break of up to five minutes if they noticed any signs of fatigue. Otherwise, they proceeded directly to the second test run.

All participants were informed that the stimuli would be presented in a randomized order from the frontal hemisphere. No feedback about source location was provided during training or testing.

### Stimuli

The stimulus was a 430 ms white-noise pulse train consisting of five 30 ms noise bursts separated by 70 ms pauses, with half-Gaussian ramps (3 ms rise time)^ 24^. To prevent monaural localization cues, level and spectral roving were applied to the stimulus as proposed by Van de Heyning et al. ^25^. For level roving, the stimulus was played back in a randomized sequence at 60, 65 and 70 dB SPL. Spectral roving was implemented by convolving the stimulus with the ipsilateral and contralateral HRTF (−90° azimuth, 0° elevation) from a public database^ 26^, resulting in a total of 66 stimuli per test run. 66 repetitions per test were considered appropriate in order to avoid fatigue effects during the experiment.

Prior to each run, participants completed a short training session with 22 stimuli (two per loudspeaker in random order at 65 dB SPL) using the response method of the following test run.

### Data analysis

Localization performance was quantified using signed bias (SB) as a measure of systematic difference between response and target (Eq. (1)) and root mean square error (RMSE) as a measure of accuracy (Eq. (2)) ^27,28^.

1 $${SB}_{k}\mathrm{=}\frac{1}{N}{\sum}_{i\mathrm{=}1}^{N}\left({r}_{k,i}\mathrm{-}{s}_{k}\right)$$

2 $${RMSE}_{k}\mathrm{=}\sqrt{\text{}\frac{1}{N}{\sum\:}_{i\mathrm{=}1}^{N}{\left({r}_{k,i}\mathrm{-}{s}_{k}\right)}^{2}}$$

$\:N$ is the number of responses per loudspeaker, $\:{r}_{k,i}$ is the $\:i$th response angle for the $\:k$th loudspeaker, $\:{s}_{k}$ is the source angle of the $\:k$th loudspeaker.

In addition to the accuracy measures, the trial duration (TD) for each input was recorded. TD was defined as the interval between stimulus onset and response confirmation, i.e. the press of the button of the LED-based method and touching the surface of the tablet.

Linear mixed-effects models were fitted for SB, RMSE, and TD. Fixed effects included response method, source angle, their interaction, and test order. The models included random intercepts for participants and random slopes where they improved model fit according to Akaike’s Information Criterion (AIC). The best-fitting models (lowest AIC) included random slopes for source angle for SB, both test order and source angle for RMSE, and test order for TD. LMMs were chosen primarily because intraclass correlation coefficient (ICC) estimates from null models indicated substantial variance attributable to participants (ICC = 0.325) and test order (ICC = 0.596) for the TD. The variance components for SB and RMSE were comparatively small (0.023 < ICC < 0.257). Nevertheless, these random effects were retained when their inclusion resulted in improved model fit, as indicated by a reduction in the AIC.

R² calculations ^29,30^ showed that fixed effect of the SB model accounted for approximately 31% (marginal R² = 0.308) of the variance, while the full model (including random effects) explained 52% (conditional R² = 0.518). The RMSE model showed a marginal R² of 0.378 and a conditional R² of 0.486. For the trial duration model, marginal R² was 0.434 and conditional R² was 0.838, indicating substantial additional variance explained by the random-effects structure.

Post-hoc comparisons were conducted on the estimated marginal means, testing pairwise differences between source-angle levels within each modality. Comparisons used 0° as the reference level and p-values were adjusted using the Holm–Bonferroni correction. Results were considered statistically significant when *p* < 0.05.

Model assumptions were assessed by examining residual distributions. Although Kolmogorov-Smirnov and Shapiro-Wilk tests indicated statistically significant deviations from normality for all three LMMs (SB: *p* < 0.01; RMSE: *p* < 0.01; TD: *p* < 0.01), visual inspection of Q-Q plots and histograms suggested minor deviations that did not substantially violate the requirements.

## Results

The analysis revealed a highly significant difference overall RMSE between the two response Methods (F(1,18) = 22.96, *p* < 0.001). Use of the LED-based method resulted in a 1.98° lower mean RMSE of 5.43° (95% CI: 4.77° to 6.10°) compared to the tablet-based method with 7.41° (95% CI: 6.75° to 8.07°). Response times showed the opposite pattern. The LED-based method resulted in a significantly longer TDs (mean 3.39 s, 95% CI: 3.14 s to 3.65 s) than the tablet-based method (mean 2.80 s, 95% CI: 2.55 to 3.05 s), corresponding to an average increase of 0.59 s per trial (F(1,16) = 25.05, *p* < 0.001).

**Fig. 3:**
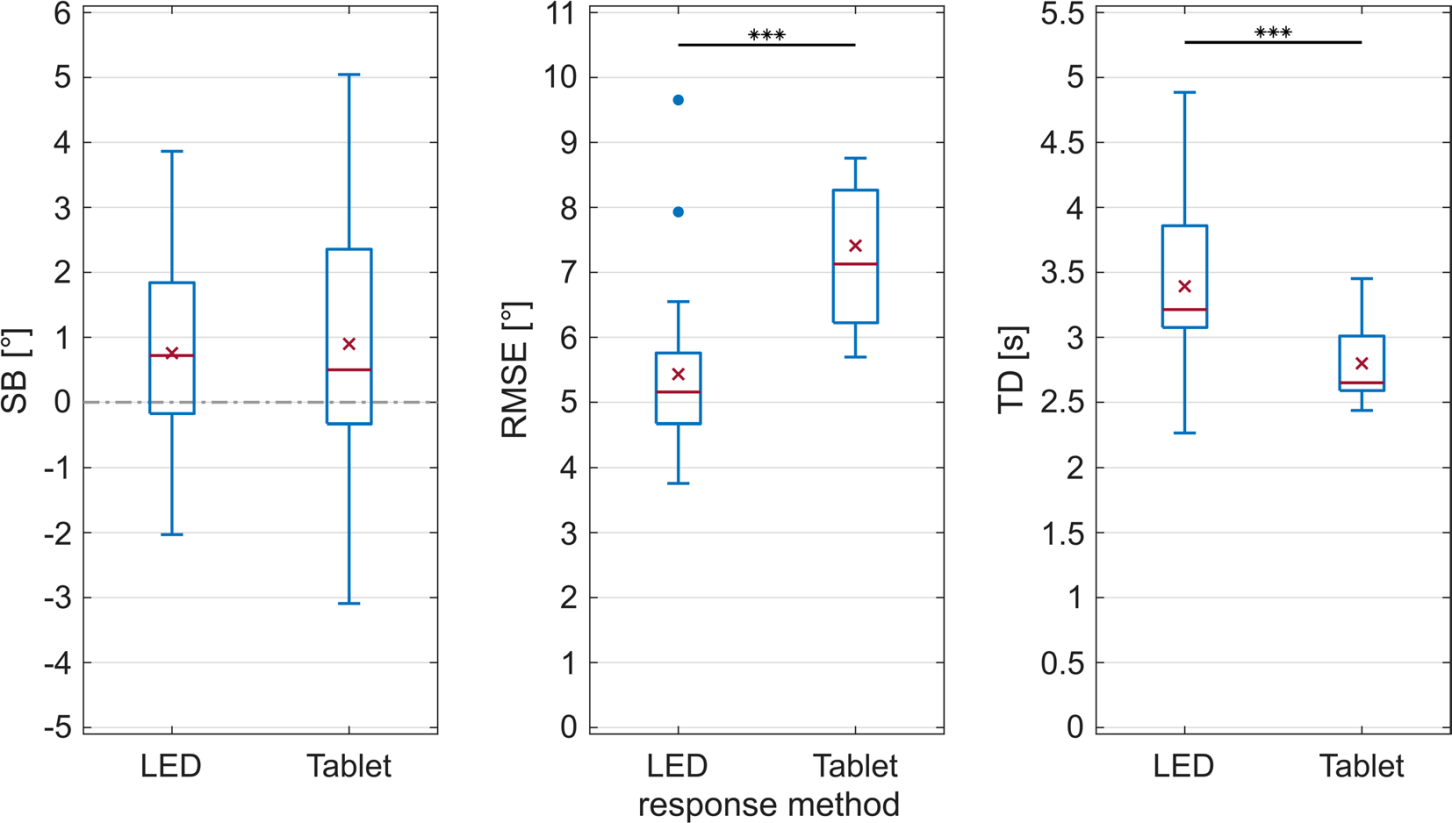
Localization test results: Boxplots display the mean signed bias (left), RMS error (middle), and response time (right) obtained with the LED-based and tablet-based response methods. The plots show no difference in overall signed bias, but highly significant differences in both overall RMSE and response time between the two methods. The Red horizontal lines indicate the median, while red crosses indicate the mean values. Black asterisks indicate significant differences between speaker positions (*p ≤ 0.05; **p ≤ 0.01; ***p ≤ 0.001).

No significant difference was observed in the SB between the two response methods (F(1, 208) = 0.14, *p* = 0.710), suggesting that both methods performed similarly in capturing the overall directional bias of participants’ localization responses (Fig. 3). Nonetheless, both methods exhibited a small rightward bias, amounting to 0.76° (95% CI: −0.03° to 1.54°) for the LED-based method and 0.90° (95% CI: 0.12° to 1.68°) for the tablet-based method.

Test order did not significantly affect SB (F(1, 208) = 2.7, *p* = 0.102), RMSE (F(1,18) = 0.003, *p* = 0.960), or TD (F(1, 16) = 2.76, *p* = 0.116), suggesting that neither learning nor carryover effects influenced the observed outcome.

### Angle-dependent Analysis

**Fig. 4:**
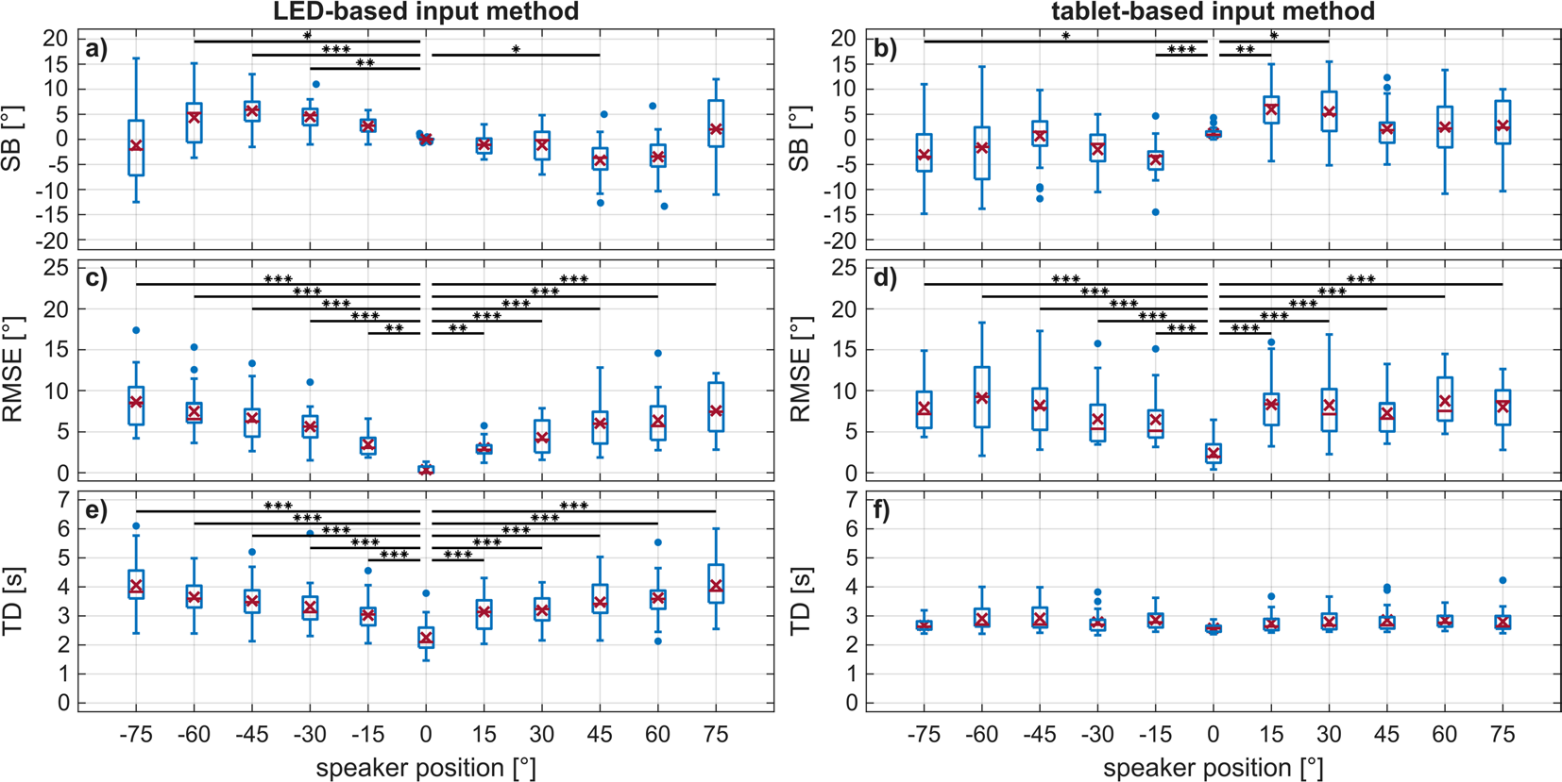
Angle dependentlocalization test results: Boxplots of the signed bias (top row), RMS error (middle row) and trial duration(bottom row) of the LED-based (left column) and tablet-based response method (right column) as afunction of speaker position. Both response methods showed minimal SB and RMSE at the midline, with reduced localizationperformance toward more lateral speaker positions. The LED-based method exhibited a systematic biastoward the frontal direction and increasing TD with greater angular distance from 0°. The tablet-basedmethod showed a more irregular SB pattern, generally higher RMSE, and comparatively stable trialdurations across speaker positions. The Red horizontal lines indicate the median, while red crossesindicate the mean values. Black asterisks indicate significant differences between speaker positions (*p≤ 0.05; **p≤ 0.01; ***p≤ 0.001).

Significant interactions between response method and source angle were observed for SB (F(20, 398) = 10.26, *p* < 0.001), RMSE (F(20, 398) = 12.22, *p* < 0.001), and TD (F(20, 356) = 15.69, *p* < 0.001). Pairwise comparisons identified the significant differences between source angles within the different modalities, as illustrated in Fig. 4. Spaghetti plots of the data (Fig. 7) indicate that the effects are relatively stable across participants. However, results of the tablet-based method exhibited generally higher between-subject variance and inter-individual differences in SB and RMSE.

#### Signed bias

In case of the LED-based method (Fig. 4 - a), the mean SB was smallest at the 0° speaker position and reached a maximum at ± 45°, followed by a decrease towards ± 75°. Bias values were predominantly positive on the left side of the array (0° to −75°) and negative on the right side (0° to 75°), indicating a systematic bias toward 0° (viewing direction), except for the most lateral position. Between-subject variance steadily increased with angular distance, rising from an IQR of 0.1° at the center to a maximum of 9.2° and 10.9° at ± 75°, suggesting higher uncertainty in localization towards more lateral positions.

The tablet-based method (Fig. 4 - b) showed a more irregular pattern. The smallest biases (0.7° to 2.1°) occurred at speaker Positions − 30°, ± 45°, −60° and 0°, whereas the largest biases (4.1° to 6.0°) were observed at speaker positions ± 15° and 30°. In contrast to the LED-based methods bias signs were reversed, with more negative values on the left and positive values on the right side of the array, indicating a systematic shift away from the center. Between-subject-variance was generally higher compared to the LED-based method and varied across speaker positions, suggesting an increased uncertainty in participants’ localization responses.

#### Root mean square error

The RMSE of the LED-based method (Fig. 4 - c) roughly followed a V-shape pattern. Mean errors increased from 0.37° at 0° to 8.65° at − 75° and 7.59° at + 75°. The Between-subject variance showed a similar trend, with variability increasing from an IQR of 0.99° at the center to 4.54° and 5.87° toward ± 75°. The data indicates more consistent localization near the center and increasing uncertainty towards the lateral positions.

The tablet-based condition (Fig. 4 - d) shows systematically higher mean errors and greater between-subject variance compared to the LED-based method. Apart from the center position with a value of 2.4° mean RMSE is high and relatively consistent, ranging between 6.5° to 9.1°. between-subject variability ranged from 4° to 7° at lateral angles and 2.26° at the midline.

#### Trial duration

TDs of the tablet-based-method (Fig. 4 - f) were relatively stable across speaker positions. Mean TDs ranged from 2.58 to 2.93 s and showed very low variability between participants (IQR = 0.21s to 0.68s), indicating fast and confident inputs regardless of the position of the sound source.

In contrast, TDs of LED-based method (Fig. 4 - e) varied considerably. Mean TDs were lowest at 0° (2.26 s) and increased steadily towards lateral positions. A linear fit revealed an increase of approximately 0.12 s per speaker position and an offset of about 0.56 s between 0° and the remaining positions. Between-subject-variance was higher compared to the tablet-based method (IQR = 0.60 s to 1.31 s) and increased slightly at the lateral positions.

**Fig. 5:**
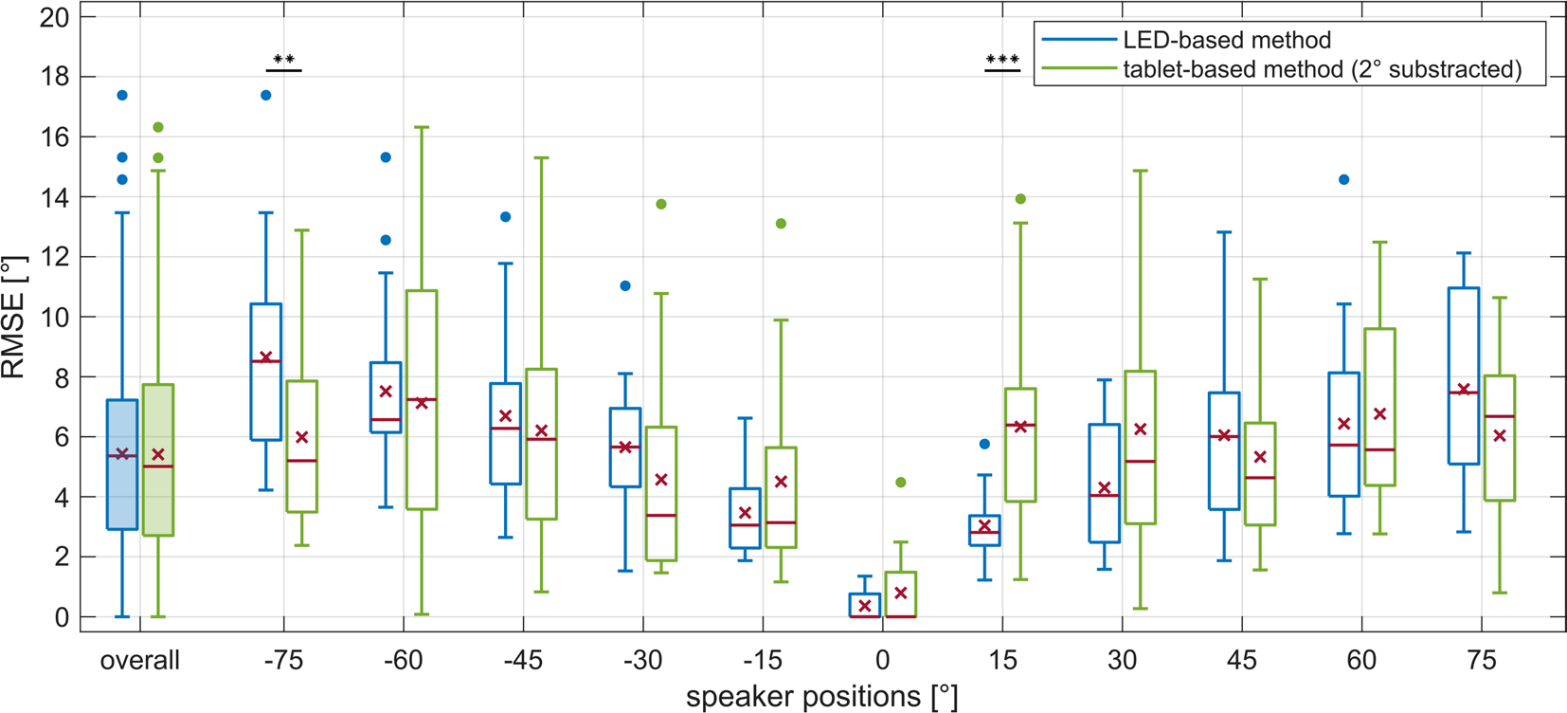
RMS error of the tablet-based response-tracking method after 2° subtraction: Boxplots show the RMSE of the LED-based methodand the RMSE of the tablet-based method after subtraction of a 2° system-level input error, plotted as afunction of speaker position. The diagram shows that, after correcting the tablet-based RMSE for the 2° input error, overall as well asangle-specific differences between the two methods are largely eliminated. The Red horizontal linesindicate the median, while red crosses indicate the mean values. Black asterisks indicate significantdifferences between speaker positions (*p≤ 0.05; **p≤ 0.01; ***p≤ 0.001).

The high between-subject-variance estimated by the null model prompted an analysis to determine whether participants, who took more time to respond also showed improved localization performance. For each source angle, we divided the SB and RMSE into responses with longer-than-average ($\:{TD}_{k}\text{}\ge\:\text{}{\overline{TD}}_{k}$) and shorter-than-average trial durations ($\:{TD}_{k}\mathrm{<}{\overline{TD}}_{k}$). The two groups were then compared using one-way ANOVA. No significant differences were found between fast and slow responses for either SB (F(1, 196) = 1.57, *p* = 0.212) or RMSE (F(1, 196) = 0.57, *p* = 0.451).

### Input accuracy

In order to determine a difference in input accuracy between the two response methods, the RMSE at 0° was compared (Table 1). The 0° input option was available as the start position on the LED strip and visually marked on the tablet, allowing for precise selection in both cases. Despite the possibility of a precise response, the results showed a significant difference (F(1, 38) = 30.95, *p* < 0.001) in the RMSE with a mean of 0.37° (95% CI: −1.60° to 0.89°) for the LED-based method and 2.41° (95% CI: 1.88° to 2.93°) for the tablet input. The results indicating a 2.0° system-level input error while using the tablet-based method, rather than a reduction in auditory localization accuracy.

**Table 1:** Comparison table summarizing characteristics of the two response tracking methods.

Characteristic	LED-based method	Tablet-based method
Frame of reference	Egocentric	Exocentric
Response Input	Rotary encoder to move lit pointer LED along the covered speaker array, confirm angle with button	Tapping the corresponding angle on a circular 2-D representation of the covered speaker array on a tablet PC
Mean signed bias	0.78°; 95% CI: −0.03° to 1.54°	0.90°; 95% CI: 0.12° to 1.68°
Mean RMS error	5.43°; 95% CI: 4.77° to 6.10°	7.41°; 95% CI: 6.75° to 8.07° ≈ 2° system-level input inaccuracy
Mean trial duration	3.39 s; 95% CI: 3.14 s to 3.65 s	2.80 s; 95% CI: 2.55 s to 3.05 s
Angle-dependent response pattern	Central tendency toward 0° likely attributable to the replay setup and methodology	Irregular bias pattern. Exploratory analysis points to method-specific overestimation of sound eccentricity

**Table 2:** Overall and angle-dependent mean and standard deviation of the signed bias [°], RMS error [°] and trial duration [s] captured with the LED- (left) and tablet-based method (right).

		LED			Tablet		
		SB [°]	RMSE [°]	TD [s]	SB [°]	RMSE [°]	TD [s]
Source angle [°]	Overall	0.76 ± 5.29	5.43 ± 3.35	3.39 ± 0.90	0.90 ± 5.96	7.41 ± 3.69	2.80 ± 0.37
	−75	−1.19 ± 7.54	8.65 ± 3.25	4.06 ± 0.96	−3.06 ± 5.44	7.99 ± 2.98	2.67 ± 0.20
	−60	4.34 ± 5.04	7.51 ± 2.88	3.65 ± 0.73	−1.68 ± 6.86	9.12 ± 4.35	2.91 ± 0.43
	−45	5.68 ± 3.36	6.70 ± 2.78	3.52 ± 0.76	0.68 ± 5.86	8.21 ± 3.74	2.93 ± 0.45
	−30	4.53 ± 2.69	5.65 ± 2.09	3.32 ± 0.81	−2.05 ± 4.12	6.57 ± 3.24	2.79 ± 0.38
	−15	2.63 ± 1.77	3.47 ± 1.36	3.02 ± 0.61	−4.06 ± 3.79	6.51 ± 3.12	2.86 ± 0.34
	0	0.05 ± 0.38	0.37 ± 0.47	2.26 ± 0.58	1.21 ± 1.08	2.41 ± 1.53	2.58 ± 0.14
	15	−1.01 ± 1.89	3.04 ± 0.97	3.14 ± 0.66	5.97 ± 4.63	8.33 ± 3.10	2.74 ± 0.32
	30	−1.14 ± 3.44	4.30 ± 1.98	3.19 ± 0.53	5.53 ± 5.62	8.26 ± 4.09	2.79 ± 0.34
	45	−4.17 ± 4.13	6.05 ± 2.97	3.47 ± 0.74	2.14 ± 4.56	7.33 ± 2.75	2.86 ± 0.45
	60	−3.47 ± 4.35	6.44 ± 2.95	3.62 ± 0.81	2.45 ± 5.72	8.76 ± 3.06	2.85 ± 0.29
	75	2.07 ± 6.47	7.59 ± 2.93	4.06 ± 0.94	2.77 ± 5.33	8.04 ± 2.64	2.80 ± 0.42

Under the assumption that this input error is similar at every point on the tablet surface, we subtracted the 2° error from the RMSE values captured with the tablet-based method. The adjusted data was then compared to the results of the LED-based method (Fig. 5), using the same LMM as before. After applying the 2° correction, the overall difference in RMSE between the two methods was no longer statistically significant. However, post-hoc comparisons with Bonferroni–Holm correction indicated that significant differences persisted at source angles − 75° (*p* = 0.004) and 15° (*p* < 0.001), with a marginally non-significant difference at 30° (*p* = 0.056).

## Discussion

The present study compared an egocentric LED-based and an exocentric tablet-based response tracking method in a free-field sound localization experiment using a concealed horizontal loudspeaker array. The two methods differ along several dimensions, including spatial reference frame (egocentric vs. exocentric), motor interaction with the input interface and visual mapping of the auditory environment. The aim of this study was to compare these methods with common measures of directional hearing. A summary of the comparison of the two methods is provided in Table 1.

Analysis of overall localization performance revealed a slight rightwards shift in the SB close to 1° for both modalities. This finding is consistent with previous reports by Seeber ^24^ and Kerber & Seeber^ 31^, who used a curtain-covered loudspeaker array and a light pointer projected onto the curtain to track participants’ responses. The observed rightward shift in the responses is likely due to the fact that the majority of participants are right-handed.

The LED-based method showed a small but significantly lower overall RMSE of 5.43° (95% CI: 4.77° to 6.10°) compared to the tablet-based method 7.41° (95% CI: 6.75° to 8.07°) and was accompanied by a 21% increase in mean TD, adding 59 s to the total measurement time for a localization test run involving 66 stimuli. However, a substantial amount of the difference, approximately 2°, can be attributed to the system level inaccuracy of tablet input. The results indicate, that comparable localizations accuracy can be achieved with both response methods, if the discrepancies between the intended target position and the finger position on the touch surface could be minimized (for example by use of a tablet pen).

The RMSE values obtained with both methods are consistent with the range of localization accuracy reported for normal-hearing listeners in previous studies. Experiments that used visible horizontal loudspeaker arrays in combination with label-based response methods and noise stimuli typically reported RMSEs between 6° and 7° ^28,32–34^. Results from studies using concealed loudspeaker arrays and alternative response methods show similar levels of accuracy. Kerber and Seeber ^31^concealed their array behind opaque acoustic fabric and used a light pointer controlled by a trackball to capture listener responses. Under these conditions, they reported an RMSE of 5.0° ± 1.8°. Likewise, Ausili et al^. 12^. conducted localization experiments in darkness using a head-tracking system and reported an RMSE equivalent response variability of 5.0° ± 1.4°.

Further angle-dependent analysis revealed systematic, modality-specific patterns that show how the response tracking method influences localization performance.

### Signed bias

Participants’ responses, measured using the LED-based method, exhibited a shift in SB toward the frontal position (0°), suggesting a systematic underestimation of the sound source eccentricity (deviation from midline). Such discrepancies between actual source position and participant responses have been previously reported in localization experiments. However, the direction and magnitude of these effects vary depending on the methodology used.

For instance, Lewald & Ehrenstein ^35^, Seeber ^24^ and Kerber & Seeber ^31^ investigated sound localization with broadband and band-pass filtered noise in normal-hearing listeners by asking them to adjust the position of a light pointer. The results varied: while Kerber & Seeber found no significant bias, Lewald & Ehrenstein, reported a general overestimation of sound eccentricity. In contrast, Seeber observed an underestimation of eccentricity when the initial laser point was set at 0° compared to a random position within ± 50°. The magnitude of underestimation increased with lateral source position, reaching approximately − 5° for a source at 50°. Although a linear trend was observed, a clear frontal bias was only evident for sound sources positioned to the left of the array. Despite the similarity in response methods across these studies, their findings do not explain the present results. One key difference may be the restriction of head movement in all three prior studies, which required participants to fixate a reference LED or track the pointer with their eyes only.

In contrast, studies that permitted head movement during or after stimulus presentation ^36,37^ or employed head-pointing as a response method ^36,38–40^ reported localization biases more comparable to those observed in the current study. The literature suggests that during head-pointing sound localization is affected by the relative position of head and trunk of the participant, resulting in a systematic shift in responses towards the median plane of the trunk ^36^.

While the effect could explain the frontal bias observed in the presented data, we recognize that our methodology is not directly comparable to the head-pointing paradigms. Although participants were free to move their head after stimulus presentation, they did so to follow the lit LED controlled via the rotary encoder, rather than to indicate the perceived sound source location through head orientation itself. Furthermore, some participants chose not to move their head during the task, instead relying solely on eye movements to track the position of the LED, suggesting that factors beyond head and eye movement may contribute to the observed effect.

We hypothesize, that this frontal bias relies on a “central tendency of judgment”^ 41^ or “central tendency in rating” ^42^. This effect describes a systematic shift in responses toward the center of the provided response scale and can be observed when participants are uncertain about their judgments. In the present study, this tendency was reflected in an increased bias toward the midline as participants’ localization uncertainty grew. However, at the most lateral loudspeaker positions (± 75°), the SB decreased and approached zero. This suggests that at extreme lateral positions, participants were able to discern that the source must be located further toward the periphery, counteracting the general tendency to respond closer to the midline.

If the central tendency in rating is the primary factor driving the (mis-)perception of the source position, a similar response pattern should be present in the data obtained using the tablet-based method. However, the distinct differences in angle-dependent responses between the two methods suggest at least one additional effect influencing the tablet-based measurements.

**Fig. 6:**
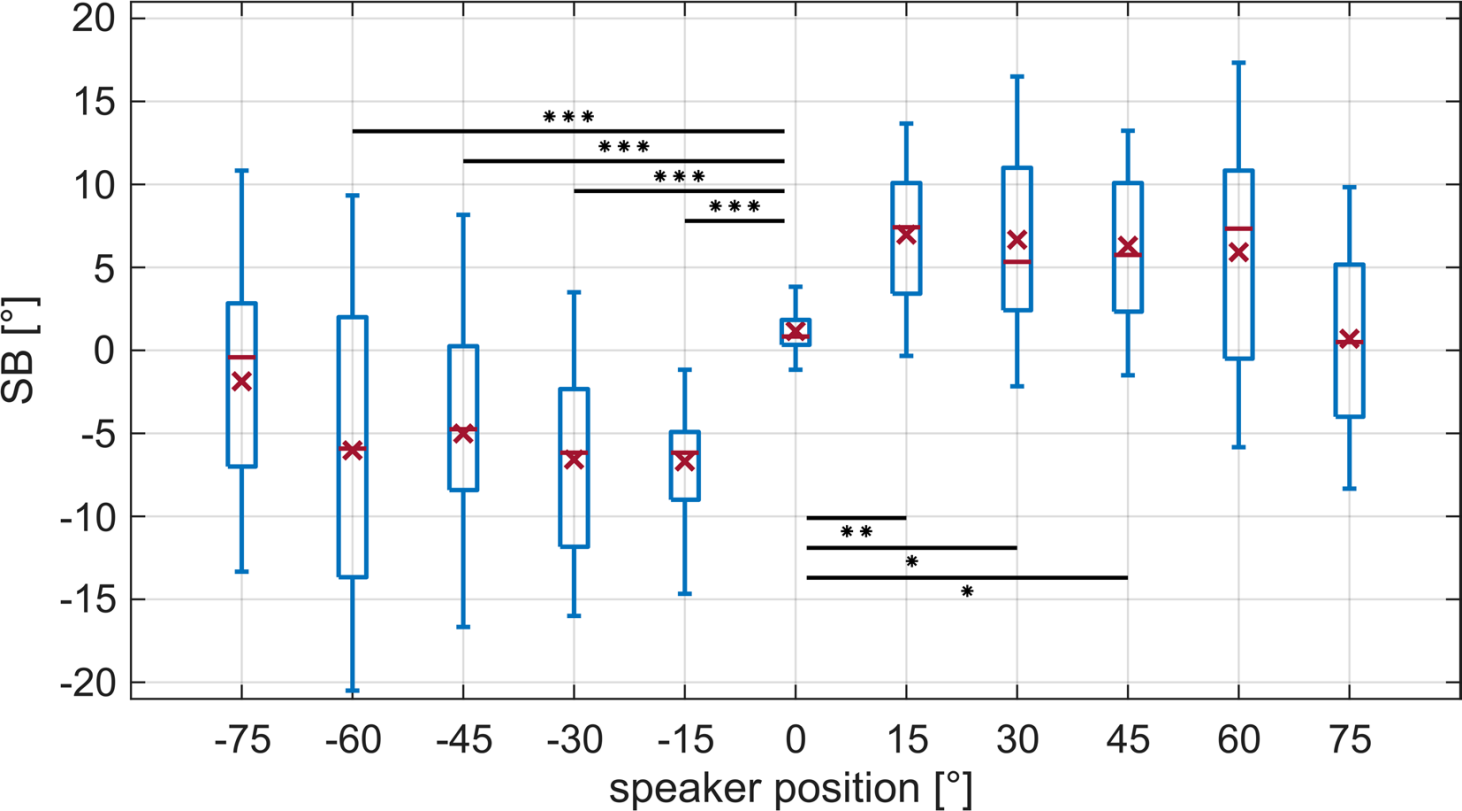
Adjusted localization biasof the tablet-based response tracking method: Boxplot of the SB [°] captured with the tablet-basedmethod as a function of speaker position. The Diagram shows the data distribution after the frontal biaswas removed by subtracting the speaker-dependent bias of the LED-based method. The process revealeda relatively constant overestimation of the sound source angle for speaker positions at ± 15° to ± 60°.The Red horizontal lines indicate the median, while red crosses indicate the mean values. Black asterisksindicate significant differences between speaker positions (*p≤ 0.05; **p≤ 0.01; ***p≤ 0.001).

In an exploratory approach we tried to isolate this potential effect by subtracting the individual angle-dependent SB observed in the LED-based method. This procedure effectively removed the frontal bias from the dataset of the tablet-based method. ANOVA analysis and post-hoc comparisons with Bonferroni-Holm correction of the adjusted data (Fig. 6) revealed a minimal mean SB at 0° and ± 75°. Intermediate angles showed a consistent offset, ranging between − 4.8° to 6.2° on the left and 5.3° to 7.4° on the right side of the array. This pattern suggests a systematic overestimation of sound source eccentricity (lateral bias) when the tablet-based method was used.

Systematic sound localization biases associated with exocentric response methods have been documented previously. Using the exocentric God’s Eye Localization Pointing (GELP) technique, Djelani et al^ 6^. reported that participants tended to point too far backward on the spherical response interface. This pattern aligns with the systematic overestimation of sound source eccentricity observed in the adjusted bias data of the present study. Djelani et al. attributed these deviations to a parallax effect arising from the spatial configuration of the response apparatus. The sphere was positioned in front of the listener, introducing a spatial offset between the body-centered auditory percept and the external response interface. From the position of the response interface the stimulus location is angled further towards the rear, resulting in the backward shift in responses (see their Fig. 7).

Similarly, Brungart et al^. 43^. utilized Styrofoam manikin heads for their GELP-based paradigm. Participants used a sensor-mounted pointer to indicate the stimulus either directly at the perceived source position or responded in relation to the reference head. Analysis of the mean azimuth error also revealed a backward bias. However, Brungart et al. turned the reference head 90°, so that its face pointed towards the right side of the participants. This modification introduces an additional transformation to the frames of reference, limiting direct comparison to the current study and other GELP configurations.

While the restriction of stimulus presentation and response inputs to the frontal hemifield could account for the observed frontal bias, and the use of an exocentric response method may contribute to the lateral bias, it is important to note that the underlying mechanisms remain speculative. Within the constraints of the current methodology, the specific causes of these systematic deviations cannot be conclusively determined.

### Root mean square error

The LED-based method showed minimal RMSE at 0° and a steady increase toward the ± 75° loudspeaker positions, indicating that participants’ localization accuracy was highest near the frontal midline. This effect is well established in the literature. Using free-field loudspeaker setups with sources placed at discrete horizontal azimuths, Stevens and Newman ^44^ showed that absolute localization error for tonal and broadband stimuli increased with angular distance from the frontal midline. Similar results have been reported in more recent studies by Yost and Xuan Zhong ^45^ and Yost ^9^, employing noise bursts, with participants indicating the perceived sound location via discrete loudspeaker selection. Consistent trends were also found by Tabry et al. ^39^. using broadband stimuli in free-field tasks involving visual feedback and a blindfolded condition, where participants reported source direction either by head or hand pointing.

This effect has been attributed to restricted head movement during stimulus presentation. Although participants in the present study were allowed to move their heads, they were instructed to keep their head oriented straight ahead during stimulus presentation and to return to this central position (indicated by a lit LED) before the start of each subsequent trial. The short stimulus duration (430 ms) further limited reactive head movements toward the sound source, resulting in testing conditions consistent to those used in the aforementioned studies.

Data collected with the tablet-based method showed similar to the LED-based method, with minimal RMSE at 0° and increasing RMSE toward ± 75°. However, overall RMSE values and between-subject variance were generally higher and fluctuated around 7.5° ± 0.5° across loudspeaker positions. After correcting for the approximately 2° system-level input error, the overall RMSE difference between the two methods was eliminated, and angle-dependent discrepancies became largely insignificant (Fig. 5). These findings suggest that the elevated RMSE observed with the tablet-based method primarily reflects limitations of the response interface rather than exocentric errors arising from remapping the auditory environment onto the two-dimensional tablet interface.

### Trial duration

While the tablet-based method showed no variation in TD across speaker positions, the LED-based method exhibited a clear increase in TD with increasing angular distance from 0°. This linear progression reflects the time required to move the LED along the strip from its initial position at 0°. Once the LED reached the approximate target location, participants required additional time to select the specific LED that best matched their perceived source position. This selection process introduced an average delay of approximately 0.56 s compared to responses at 0°, where no prior LED movement was necessary.

**Fig. 7:**
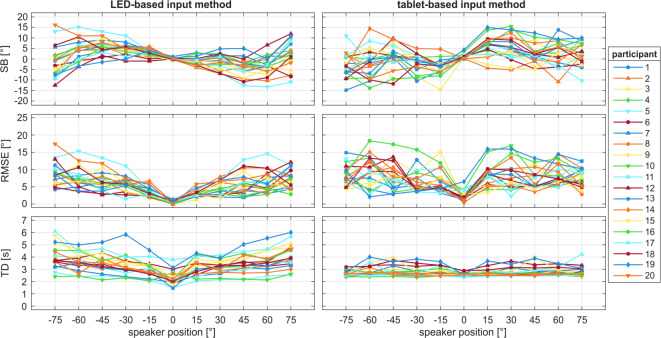
Angle dependent localization test results for every participant: Spaghetti plots of the individual signed bias (top row), RMS error (middle row) and trial duration (bottom row) captured with the LED-based (left column) and tablet-based response method (right column) as a function of speaker position. The plots show the stability of the relationship between the parameters and the source position on an individual level.

Notably, the LED-based method revealed greater variability in TDs across participants (Fig. 7). Pointing to a location on the tablet surface appeared to be a natural, fast, and consistent process, whereas the LED-based method, though intuitive, showed differences in individual utilization. Some participants took their time to precisely position the LED, while others made only rough adjustments, quickly placing the LED near the perceived source location without fine-tuning. Despite these variations in response strategies there was no significant difference in SB or RMSE when comparing the group with slower to the group with faster than average TDs.

## Conclusion

This study compared an egocentric LED-based and an exocentric tablet-based response tracking method for sound localization measurements with a concealed horizontal loudspeaker array.

Overall, the LED-based method showed a significantly lower RMSE, whereas the tablet-based method enabled shorter trial durations. The findings are consistent with previous reports using similar response methods and are comparable to results obtained with technically more complex approaches, such as head tracking. Importantly, a substantial portion of the difference in RMSE could be attributed to a system-level input error of the tablet-based method, arising from discrepancies between the intended target position and the actual finger position on the touchscreen.

Angle-dependent analysis, revealed systematic, modality-specific effects. Both methods exhibited a central bias and reduced accuracy at more lateral positions. Exploratory analyses of the tablet-based method suggested an additional lateral bias, which may reflect cognitive remapping of the auditory environment onto the tablet interface.

These findings demonstrate that the choice of response method can systematically affect participant responses and should be carefully considered in the design of sound localization tests.

## Supplementary Information

Below is the link to the electronic supplementary material.

Supplementary Material 1

## Data Availability

The authors are open to sharing materials to support the development of similar sound localization setups in other research labs or clinical settings. Source code for the response tracking applications are available upon reasonable request. Interested parties are encouraged to contact the authors directly for access.
